# Integrating Self-Organizing Maps, Positive Matrix Factorization and Time-Series Decomposition for Urban Air Pollution Source Apportionment: A Comparative Study of Bulgarian Cities

**DOI:** 10.3390/molecules31101725

**Published:** 2026-05-19

**Authors:** Stefano Fornasaro, Pierluigi Barbieri, Reneta Dimitrova, Sabina Licen, Stefan Tsakovski

**Affiliations:** 1 Department of Chemical and Pharmaceutical Sciences, University of Trieste, Via Giorgieri 1, 34127 Trieste, Italy; sfornasaro@units.it (S.F.); barbierp@units.it (P.B.); 2 Department of Meteorology and Geophysics, Faculty of Physics, Sofia University “St. Kliment Ohridski”, 1164 Sofia, Bulgaria; r.dimitrova@phys.uni-sofia.bg; 3 Chair of Analytical Chemistry, Faculty of Chemistry and Pharmacy, Sofia University “St. Kliment Ohridski”, 1164 Sofia, Bulgaria

**Keywords:** air pollution, SOM, PMF, TSA

## Abstract

Receptor modeling of ambient pollutant concentrations plays a central role in urban air quality assessments. This study proposes an integrated framework combining Self-Organizing Maps (SOM), Positive Matrix Factorization (PMF), and Time-Series Analysis (TSA) for a comprehensive evaluation of urban air pollution patterns and source dynamics. The methodology was applied to multi-annual air quality and meteorological datasets (2009–2018) from two major Bulgarian cities, Plovdiv and Varna. The SOM was used for assessing the overall parameter patterns of the cities, leading to a clear clustering of the site samples on the map. Thus, PMF was run separately for the two sites, identifying a different number of sources (three and four, respectively). Traffic-related and sulfur-rich combustion sources were identified in both cities, while a crustal/resuspended dust factor was observed only in Varna. TSA revealed distinct temporal behaviors among source types. Traffic-related aerosol contributions decreased in both cities (−5.14% yr^−1^ in Plovdiv; −9.30% yr^−1^ in Varna), whereas sulfur-rich combustion factors showed increasing trends (+4.64% yr^−1^ and +2.97% yr^−1^, respectively). Traffic fresh exhaust factors exhibited pronounced seasonal variability and significant weekday–weekend differences in both cities. The integrated SOM–PMF–TSA framework enhanced source interpretability and temporal characterization, providing a robust approach for urban air quality assessment and supporting targeted air pollution management strategies.

## 1. Introduction

Air pollution remains a major environmental and public health concern, with impacts expected to persist over the coming decades. Recent estimates from the United Nations indicate that 58% of the world’s population currently lives in urban areas, with projections suggesting an increase to approximately 67% by 2050 [1]. Urban environments concentrate both emission sources and exposed populations, leaving most residents subjected to pollutant levels exceeding recommended guidelines [2]. In Bulgaria, urbanization is already high (approximately 74%) [1], further emphasizing the importance of urban air quality management. The national air quality monitoring network in Bulgaria is integrated into an information system responsible for real-time air quality reporting in accordance with EU directives. This monitoring is carried out by automatic monitoring stations (AMS) that primarily record concentrations of major pollutants alongside a limited set of meteorological variables. However, the use of monitoring data remains largely confined to air quality reporting, while air quality assessment modeling studies, critical for informing environmental authorities, are rarely undertaken.

Receptor modeling of ambient pollutant concentrations plays a central role in urban air quality assessment, as it enables quantitative identification and apportionment of emission sources directly from monitoring data [3]. This capability is particularly important in studies addressing health impacts and policy design, where understanding source-specific contributions is essential for prioritizing mitigation measures and reducing population exposure [4].

The Self-Organizing Map (SOM) algorithm has been used in several studies for assessing air quality. The benefit of using SOM is that it can easily handle big datasets, extracting recurrent pollutant patterns from the data. It has been used, usually coupled with k-means clustering, for assessing temporal and spatial pollutant patterns [5,6,7,8], and, in some studies, for source identification [9,10,11]. In recent studies, a combined approach using SOM and Positive Matrix Factorization (PMF) proved to be useful for taking advantage of the benefits provided by the two algorithms. Hassan et al. used PMF for assessing pollution sources and SOM for establishing the correlation between the sources and meteorological and population characteristics [12]. Liu et al. used SOM for classifying the daily atmospheric motion and PMF for source apportionment of PM_2.5_ [13]. The merged results allowed them to evaluate the induced direct radiative effects over a region. Fornasaro et al. used PMF to independently confirm the pollutant signature obtained using SOM coupled with HC (hierarchical clustering) analysis [14]. Biondi et al. applied both SOM and PMF for assessing pollution sources in oil and gas extraction areas and discussed the benefits and drawbacks of both techniques with respect to the aim of their study [15]. Buweihaidiqie Maitituersun et al. used SOM for visualizing the spatial distribution of pollution patterns related to atmospheric dustfall fluxes [16]. PMF was used to quantify the contribution of diverse heavy metals to the atmospheric dustfall fluxes. Finally, the consistency and complementarity of the two model results were checked.

The combined application of PMF and time-series analysis in air quality studies remains relatively limited, although this integrative framework provides clear methodological advantages compared with the use of either approach independently. Temporal pattern analysis can support and validate PMF factor interpretation, thereby strengthening confidence in source identification [17,18,19,20]. In particular, the evaluation of seasonal variability may refine factor characterization, as apparent discrepancies among different investigations may reflect temporal modulation of the same emission source rather than fundamentally distinct sources [17]. To date, most time-series applications linked to PMF have focused on diurnal [18,19,21], weekly [19], and seasonal profile analyses [17,19]. More advanced analytical extensions include Fourier analysis of PMF-derived source contributions [22] and cross-correlation analysis between factor contributions in time [23]. However, to the best of our knowledge, no air quality study has yet combined PMF with formal time-series decomposition explicitly separating long-term trend and seasonal components of source contributions, thereby providing a more comprehensive assessment of their temporal dynamics. The suitability of TSA for air quality applications lie in the characteristics of regulatory monitoring datasets, which typically consist of long, continuous, and standardized time series, while often lacking detailed meteorological variables (e.g., wind direction), making decomposition-based approaches particularly appropriate for isolating trend and seasonal components of source contributions.

In view of the demonstrated complementarity between SOM and PMF, and the recognized potential of time-series analysis for strengthening source interpretation, the present study aims to propose and implement an integrated air quality assessment workflow combining SOM, PMF, and time-series decomposition analysis (TSA). By integrating unsupervised pattern recognition, receptor modeling, and temporal signal separation within a unified analytical scheme, the study seeks to enhance source identification robustness, improve interpretation of seasonal and inter-annual variability, and provide a more comprehensive evaluation of urban air quality dynamics. This workflow is intended as a transferable methodological approach for urban environments characterized by complex emission mixtures and evolving anthropogenic pressures. The integrated methodological framework has been applied to air quality mandatory monitoring datasets from two of the largest Bulgarian cities.

## 2. Results

**Figure 1:**
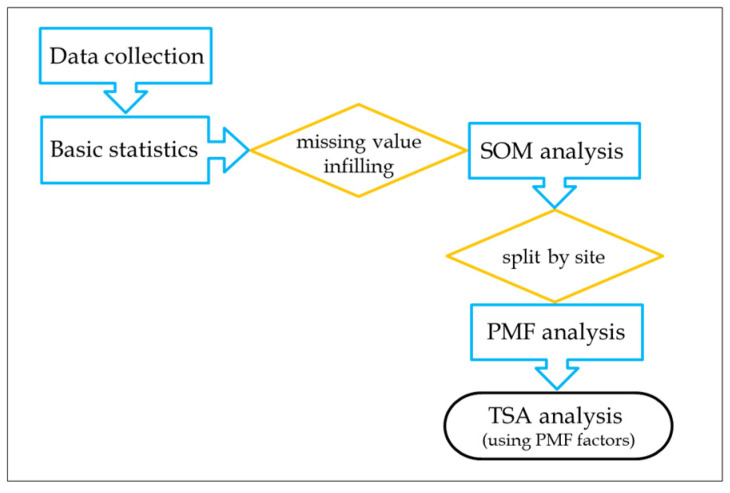
Data analysis scheme.

Several data analysis steps were performed on the collected data (Figure 1). Firstly, basic statistical parameters were evaluated. Afterwards, further elaboration algorithms were selected, and thus we decided to perform data infilling of missing data. The infilled data were used to build a SOM model and, by interpreting its outcomes, we decided to split the data by site for further elaboration with the PMF algorithm. Finally, the PMF model outputs (factors) were used to perform TSA.

### 2.1. Basic Statistics

Daily mean concentrations of PM_10_, NO, NO_2_, SO_2_, and CO (µg/m^3^) and meteorological variables were analyzed for the urban background monitoring stations in Plovdiv and Varna over the period of 2009–2018. Descriptive statistics for each variable and site are presented in Table 1.

All variables exhibited non-normal distributions with pronounced dispersion between the 25th and 75th percentiles, justifying the application of non-parametric statistical tests. Missing data were limited (<3% for all variables), indicating high dataset completeness.

For all investigated pollutants, median concentrations were consistently higher in Plovdiv compared to Varna. The largest differences were observed for PM_10_ and NO_2_. The median PM_10_ concentration in Plovdiv (37.15 µg/m^3^) exceeded that in Varna (25.26 µg/m^3^), while median NO_2_ levels were 21.24 µg/m^3^ and 13.09 µg/m^3^, respectively. Similarly, SO_2_ and CO concentrations were significantly elevated in Plovdiv.

The Wilcoxon rank-sum test confirmed statistically significant inter-site differences for CO (*p* < 0.001), NO (*p* < 0.05), NO_2_ (*p* < 0.001), PM_10_ (*p* < 0.001), and SO_2_ (*p* < 0.001). In contrast, air temperature did not differ significantly between the two cities (ns). Relative humidity and wind speed were significantly higher in Varna compared to Plovdiv (*p* < 0.001).

The variability of pollutant concentrations was more pronounced in Plovdiv, as indicated by higher mean values and wider interquartile ranges, particularly for PM_10_ and nitrogen oxides. The mean PM_10_ concentration in Plovdiv (47.83 µg/m^3^) exceeded that in Varna (29.76 µg/m^3^) by approximately 60%. Similar patterns were observed for NO_2_ and SO_2_.

Meteorological parameters showed comparable temperature distributions between the two sites; however, Varna exhibited consistently higher relative humidity and wind speed, reflecting its coastal climatic influence.

Compliance with air quality standards was evaluated using limit values defined in Directive 2008/50/EC on ambient air quality and cleaner air for Europe (EU standards) and guideline values from the World Health Organization (WHO) Air Quality Guidelines. Exceedance statistics are summarized in Tables S1–S5.

Annual mean PM_10_ concentrations were assessed against the EU annual limit value of 40 µg/m^3^ (Table S1). At the Plovdiv station, the annual mean PM_10_ concentration exceeded the EU limit value of 40 µg/m^3^ during 2009–2013, and again in 2016–2017. The highest annual mean was recorded in 2011 (60.54 µg/m^3^). In contrast, Varna did not exceed the annual PM_10_ limit in any year of the studied period, with annual means consistently below 40 µg/m^3^. Regarding the daily limit value (50 µg/m^3^, not to be exceeded more than 35 times per year), Plovdiv exhibited substantial non-compliance throughout the study period (Table S2). The number of exceedance days ranged from 44 (2014) to 162 (2009), consistently surpassing the regulatory threshold in all years. Varna also exceeded the permitted 35 days in several years (2009–2011 and 2014), with a maximum of 87 exceedance days in 2010, while compliance was achieved in the remaining years.

Overall, PM_10_ non-compliance was markedly more pronounced and persistent in Plovdiv than in Varna.

Annual mean NO_2_ concentrations remained below the EU limit value of 40 µg/m^3^ at both sites throughout the study period (Table S3). In Plovdiv, annual means ranged from 20.23 to 27.15 µg/m^3^. In Varna, values varied more substantially (4.36–32.98 µg/m^3^), with the highest level recorded in 2018, yet still being below the regulatory threshold. However, exceedances of the WHO daily guideline value for NO_2_ (25 µg/m^3^) were frequent (Table S4). In Plovdiv, the number of exceedance days ranged from 76 (2018) to 168 (2009), indicating persistent non-compliance with the WHO recommendation. Varna showed considerable inter-annual variability, with high exceedance frequencies in 2009–2011 and 2018 (up to 205 days in 2009 and 202 exceedance days in 2018), whereas full compliance was observed in 2013 and 2016.

Thus, while annual NO_2_ levels complied with EU standards, short-term exposure frequently exceeded WHO guideline values at both sites.

Daily SO_2_ concentrations remained under the EU regulatory threshold of 125 µg/m^3^ but consistently exceeded the WHO health-based guideline of 40 µg/m^3^ at both sites (Table S5). In Plovdiv, exceedances were recorded primarily during 2009–2013, with a peak of 56 days in 2011. No exceedances were observed after 2014. Varna exhibited very few exceedances, with sporadic occurrences between 2009 and 2013 and complete compliance from 2014 onwards.

The data indicate a clear improvement in SO_2_ air quality over the studied period at both sites.

### 2.2. Missing Data Infilling

Considering an overall number of rows of 7304, 198 rows (2.7%) were found to contain at least one “not available” value. The overall number of “not available” values was 292 out of 36520 (0.8%). In Figure S1, the not available data distribution is presented. The data infilling was performed separately by site, using an algorithm that considers the time series (see Section 4.2.2 for details).

### 2.3. SOM Analysis

**Figure 2:**
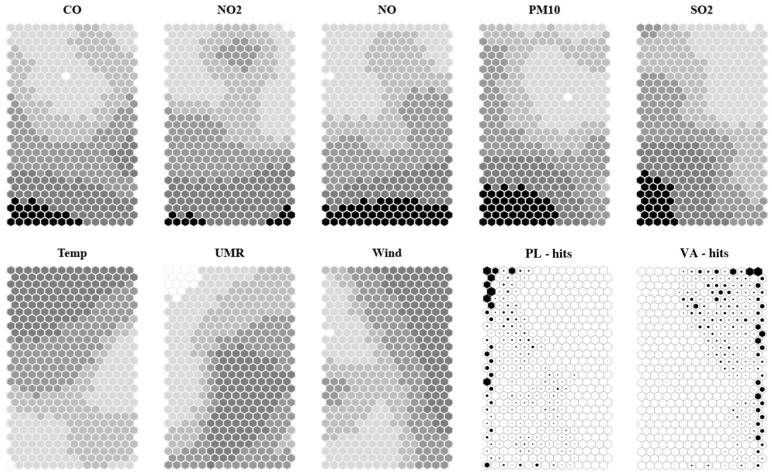
Heatmaps for each variable used for SOM training; the darker the filling, the higher the value. The grayscale spans from “LowerOut” to “UpperOut” values in Table S7. White color is for values < “LowerOut” and black color for values > “UpperOut”; hits for Plovdiv site; hits for Varna site. In hit plots, the greater the filling, the higher the number of samples represented by the neuron.

SOM is an unsupervised analysis technique, and was used for assessing possible sample grouping and variable correlation, giving the algorithm non-*a priori* knowledge about the site splitting. Z-score normalization was applied before SOM training. SOM analysis was performed on the whole dataset, comprising five pollutant variables and three meteorological variables (7304 × 8). Two different maps were trained, one “small” and one “regular” (see Section 4.2.2 for details). The square root of the first two eigenvalues of the dataset, used for selecting the map dimension ratio, was 1.6. All the maps were initialized, starting from the first two eigenvectors of the dataset. The small map was composed by 13 × 8 neurons, with a map dimension ratio of 1.6, and it was trained for two epochs. The QE, TE, and DME map quality values were 1.48, 0.033, and 0.50, respectively. The regular one was composed by 29 × 15 neurons, with a dimension ratio of 1.9, and it was trained for four epochs. The QE, TE, and DME map quality values were 1.24, 0.032, and 0.38 respectively. Thus, we decided to further investigate the results obtained by the regular map, which presented better quality values. In Figure 2, the modeled value distribution on the map for each variable (heatmap) is shown. Each neuron is represented by a hexagon. The heatmap values are represented in grayscale, with extremely low values in white and extremely high values in black. Basic statistics for the modeled values as well as details about the grayscale building are reported in Table S6. In Figure 2, the distribution of the samples (hits) on the map, split by site, are represented too. The more the samples represented by a neuron, the more the filling of the corresponding hexagon. In Figure S2 and Table S7 the percentage of samples for each site, represented by each neuron, is reported.

The heatmaps presented in Figure 2 show that, generally, in the lower part of the map, neurons represent highly polluted samples, whereas, in the upper part, they represent background air (low polluted). The distribution through the map is similar for CO, NO, and NO_2_, which show a gradient from the upper-left part of the map to the lower part. PM_10_ and SO_2_ gradients start from the upper right part of the map to the lower left. Temperature higher values are represented in the upper part of the map while lower values are in the lower part. Relative humidity values rise from the upper-left to the lower-right part of the map. Wind speed values are higher in the right part of the map with respect to the left.

The site hits on the SOMs of the two sites represented in Figure 2 clearly show that there is a different distribution pattern for the samples belonging to different sites. Varna samples are gathered in the right part of the map and the upper right corner of the map, which represent rather low pollution, high wind speed, and rather high relative humidity. Plovdiv is most represented on the left and upper-left sides of the map, with a spot in the lower left corner characterized by high values of all the pollutants. The left side of the map is characterized by rather high values of PM_10_ and SO_2_, low relative humidity, low wind speed, and high temperature.

Considering that the algorithm had no previous knowledge about the site assignment of the samples, we can conclude that the sites presented different fingerprints related both to pollutants and to meteorological parameters. Thus, we decided to split the data by site for further investigation.

### 2.4. PMF Analysis

The optimal number of factors was determined through a multi-criteria approach, evaluating mathematical stability alongside physical interpretability (Table S8). For Plovdiv, a three-factor solution was identified as the most robust. While the two-factor solution was stable (100% BS mapping), the Q/Q_exp_ ratio of 1.55 indicated that the model failed to account for significant variance in the data. Increasing the solution to three factors yielded a Q/Q_exp_ of 0.94, aligning closely with the theoretical ideal and suggesting a near-optimal fit. Although the four-factor solution further reduced the Q value, it resulted in factor splitting and instability, evidenced by a drop in BS mapping to 70% for one factor. Consequently, the three-factor solution was selected for its superior balance between mathematical fit and reproducibility. For Varna, the four-factor solution was chosen over the three-factor alternative based on improved diagnostic performance. The transition from three to four factors resulted in a notable decrease in the Q/Q_exp_ ratio from 2.60 to 2.06, indicating a more precise capture of the complex source profiles at this site. Crucially, the four-factor solution demonstrated perfect reproducibility, with 100% BS mapping for all factors, compared to 95% in the three-factor case. For both sites, DISP analysis showed no swaps, and residuals remained randomly distributed (mostly within ±3 standard deviations), confirming that the selected solutions were not influenced by rotational ambiguity or significant modeling errors.

**Figure 3:**
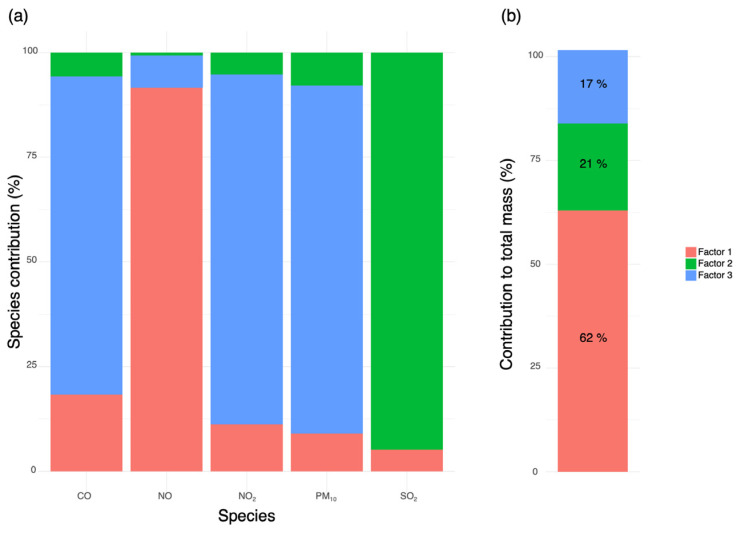
PMF-derived source profiles and relative factor contributions for Plovdiv: (**a**) species contribution profiles (in %) of the identified PMF factors for the analyzed pollutants; (**b**) relative contribution (in %) of each PMF factor to the total pollutant mass. Factor 1 was associated with fresh traffic exhaust emissions, Factor 2 with sulfur-rich stationary combustion sources, and Factor 3 with aged traffic-related aerosols.

For Plovdiv (Figure 3), three factors were identified, contributing 62%, 21%, and 17% to the total pollutant mass, respectively. The profiles were clearly differentiated in terms of dominant species. Factor 1 was strongly dominated by NO, with only minor contributions from CO, NO_2_, PM_10_, and SO_2_, indicating primary traffic emissions (fresh exhaust). Factor 2 exhibited a pronounced dominance of SO_2_, while all other species contribute marginally, suggesting sulfur-rich stationary combustion sources. Factor 3 was defined by substantial contributions of NO_2_, PM_10_, and CO, with comparatively low NO and negligible SO_2_, consistent with traffic-related emissions with a higher proportion of oxidized nitrogen species and particulate matter.

**Figure 4:**
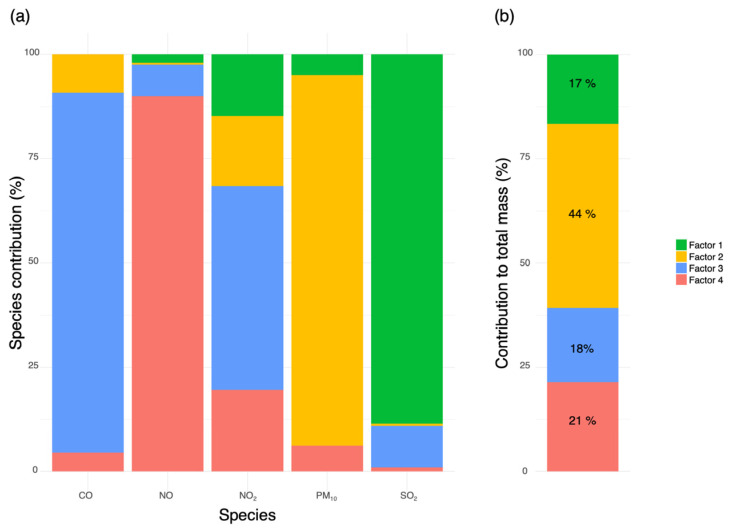
PMF-derived source profiles and relative factor contributions for Varna: (**a**) species contribution profiles (in %) of the identified PMF factors for the analyzed pollutants; (**b**) relative contribution (in %) of each PMF factor to the total pollutant mass. Factor 1 was associated with sulfur-rich combustion sources, Factor 2 with crustal/resuspended particulate matter, Factor 3 with aged traffic-related aerosols, and Factor 4 with fresh traffic exhaust emissions.

For Varna (Figure 4), four factors were identified, contributing 17%, 44%, 18%, and 21% to the total pollutant mass, respectively, with similarly well-differentiated profiles. Factor 1 was characterized by a strong dominance of SO_2_, with only minor contributions from NO_2_ and PM_10_, indicating sulfur-rich combustion sources. Factor 2 represented the largest mass contribution and was dominated by PM_10_, accompanied by smaller contributions from NO_2_ and CO, suggesting a particulate-driven source profile. Factor 3 was characterized by substantial contributions of CO and NO_2_ with negligible PM_10_, consistent with traffic-related emissions. Factor 4 is overwhelmingly dominated by NO, with a secondary contribution from NO_2_ and minimal influence from other species, reflecting primary NO-rich emissions.

### 2.5. Time-Series Analysis of PMF Source Contributions

Time-series analysis was applied to the PMF factor contributions to quantify long-term trends, seasonal behavior, and weekday–weekend differences. The results for the three resolved factors for Plovdiv are summarized below, while corresponding time-series analysis plots for Varna are presented in the Supplementary Materials (Figures S5–S8). It should be noted that the described trends refer to the relative contribution of factors and therefore reflect changes in the relative importance of sources within the PMF solution, which would provide directional insight into changes in absolute emissions.

**Figure 5:**
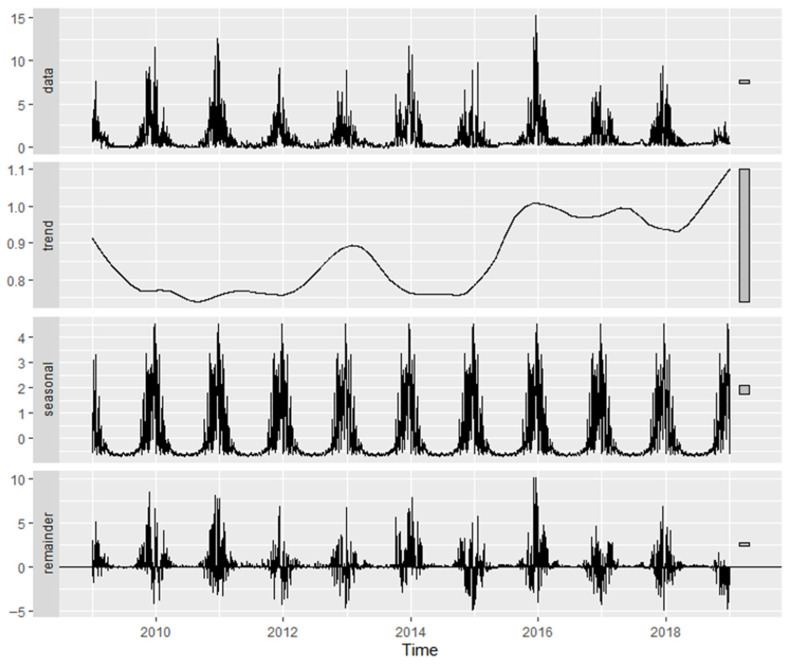
Time-series decomposition of PMF Factor 1 contributions in Plovdiv, including long-term trend, seasonal component, and residuals.

Figure 5 presents the time-series decomposition of Factor 1 (fresh exhaust) in Plovdiv, illustrating the observed contributions together with the extracted long-term trend, seasonal component, and residual variability.

A statistically significant upward trend was identified (Mann–Kendall (MK) *p* < 2.2 × 10^−16^), with Sen’s slope equal to +2.43% yr^−1^. Monthly differences were highly significant (Kruskal–Wallis *p* < 2.2 × 10^−16^), and this factor exhibited the strongest seasonal component among the three, with a seasonal strength of 33.77%. Although the Quasi-Seasonal (QS) test did not indicate significant seasonal asymmetry (*p* = 1), a highly significant weekday–weekend effect was observed (Wilcoxon *p* = 4.31 × 10^−15^).

**Figure 6:**
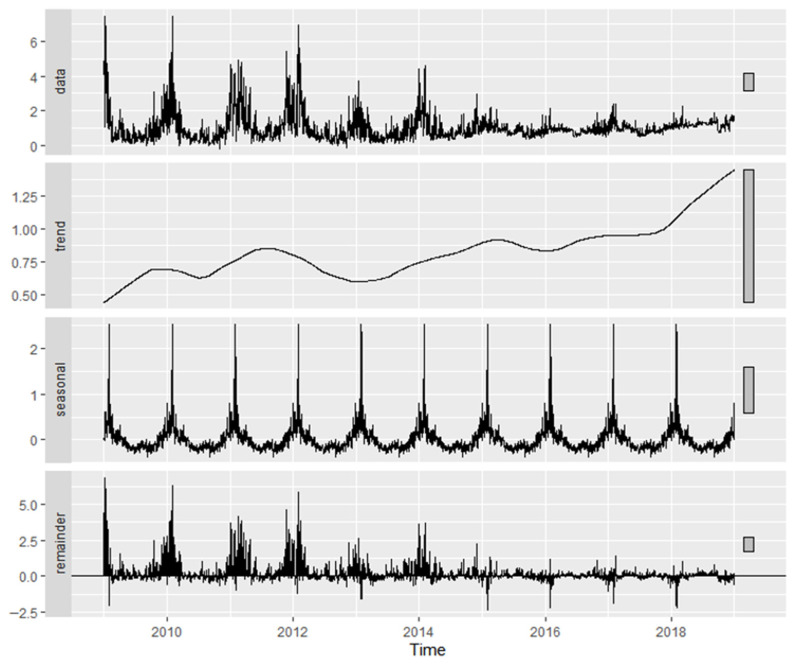
Time-series decomposition of PMF Factor 2 contributions in Plovdiv, including long-term trend, seasonal component, and residuals.

The decomposition of Factor 2 (sulfur-rich combustion), shown in Figure 6, demonstrated a statistically significant positive trend (MK *p* < 2.2 × 10^−16^), corresponding to an annual increase of +4.64% yr^−1^ according to Sen’s slope. Monthly variability was also highly significant (Kruskal–Wallis *p* < 2.2 × 10^−16^), and seasonal strength was estimated at 18.58%. The QS test did not indicate significant seasonal asymmetry (*p* = 1). Furthermore, no statistically significant weekday–weekend difference was detected (Wilcoxon *p* = 0.641).

**Figure 7:**
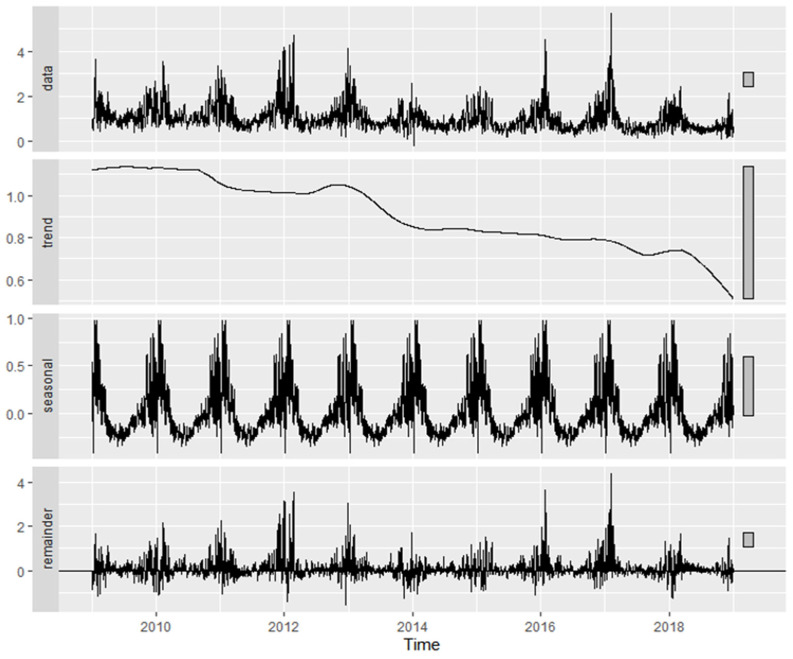
Time-series decomposition of PMF Factor 3 contributions in Plovdiv, including long-term trend, seasonal component, and residuals.

Factor 3 (traffic-related aerosol), shown in Figure 7, exhibited a statistically significant decreasing trend (MK *p* < 2.2 × 10^−16^), with Sen’s slope indicating a decline of −5.14% yr^−1^. Monthly variability was highly significant (Kruskal–Wallis *p* < 2.2 × 10^−16^), and seasonal strength reached 24.35%, reflecting pronounced intra-annual modulation. The QS test confirmed significant seasonality (*p* = 0.002), and a significant weekday–weekend difference was detected (Wilcoxon *p* = 7.4 × 10^−6^).

Table 2 summarizes the temporal characteristics of the PMF-derived factors in Plovdiv and Varna. Comparison of the common source types reveals both similarities and differences in their temporal behavior. The traffic (fresh exhaust) factor (F1 in Plovdiv; F4 in Varna) shows a consistent increasing trend in both cities (+2.43% yr^−1^ and +1.03% yr^−1^, respectively), accompanied by relatively strong seasonal components (33.77% in Plovdiv and 27.21% in Varna) and statistically significant weekday–weekend differences. The sulfur-rich combustion factor (F2 in Plovdiv; F1 in Varna) also exhibits increasing trends in both locations (+4.64% yr^−1^ and +2.97% yr^−1^, respectively), but with weaker seasonal variability, particularly in Varna (8.19%), and no significant weekday–weekend signal. The temporal behavior in Varna (Figure S5) is consistent with relatively stable combustion-related contributions and aligns with reported episodic exceedances of SO_2_ and particulate emissions from industrial sources in the Varna–Devnya region during the studied period, including documented EU regulatory sanctions in 2017. In contrast, the traffic-related aerosol factor (F3 in both cities) shows a decreasing trend in both Plovdiv (−5.14% yr^−1^) and Varna (−9.30% yr^−1^), combined with moderate to strong seasonal variability (24.35% and 25.20%, respectively) and significant weekday–weekend differences. The crustal/resuspended dust factor (F2) identified only in Varna exhibits a weak negative trend (−1.68% yr^−1^) and minimal seasonal variability (6.46%), while still showing a statistically significant weekday–weekend signal. Notably, this is the only factor for which the weekend median exceeds the weekday median. This behavior distinguishes it from the other factors, which generally display stronger seasonal components and more consistent trend patterns.

## 3. Discussion

The combined application of basic statistical analysis, Self-Organizing Maps (SOM), Positive Matrix Factorization (PMF), and time-series analysis (TSA) provides a multi-layered interpretation of urban air quality, linking concentration variability, pattern recognition, source apportionment, and temporal dynamics. While basic statistics captured overall variability, the SOM analysis reveals structured patterns in the data space, which were further resolved into interpretable source contributions by PMF and temporally contextualized by TSA.

The SOM results demonstrated that the variability in pollutant concentrations was not random but organized into distinct clusters reflecting underlying emission regimes. Such pattern recognition approaches have been widely applied in environmental studies to identify non-linear relationships and hidden structures in complex datasets [24,25]. In the present case, SOM clustering supports the PMF-derived factor separation, confirming the presence of dominant functional categories such as traffic-related emissions, sulfur-rich combustion, and particulate-associated sources. The consistency between SOM patterns and PMF factors strengthens the robustness of the source apportionment results and reduces ambiguity in factor interpretation.

PMF is traditionally the most widely used and reliable method for source apportionment. However, some drawbacks are present when computing PMF models, such as the possible troubles in defining the optimal number of factors, or in reliably defining the experimental uncertainties, especially when data are collected with real-time instruments [4]. One of the potential limitations of this study is the reliance on secondary air quality data, which precluded a direct, first-hand validation of sampling and analytical uncertainties. To address the absence of specific metadata, a conservative, standardized estimation protocol was adopted. The high stability of the solutions, evidenced by 100% BS mapping and the absence of DISP swaps, confirmed that the identified factors (three for Plovdiv and four for Varna) are statistically significant and not artifacts of the uncertainty estimation. Another limitation relates to the interpretation of PMF factors derived from the constrained air pollutant dataset used in this study. The absence of key chemical tracers (e.g., volatile organic compounds, ions, metals) and relevant meteorological variables may constrain source discrimination and increase interpretative uncertainty, thereby raising the potential for overinterpretation of PMF-derived source assignments.

PMF analysis identified comparable source types in both cities, with traffic emissions and combustion-related sources dominating the pollutant profiles. This finding is consistent with receptor modeling studies across European urban environments, where similar factors (traffic exhaust, secondary or mixed traffic aerosols, and combustion sources), are commonly reported [26,27]. More recent studies further confirm the robustness of PMF in resolving urban emission sources while emphasizing the need for cautious interpretation of temporal dynamics and source variability [28,29]. The differentiation between fresh traffic emissions (NO-dominated) and traffic-related aerosols (NO_2_–PM_10_–CO mixtures) aligns with established observations that urban traffic contributions can be separated into primary and more processed components.

A key distinction between the two cities was the presence of a crustal/resuspended dust factor in Varna, which was not observed in Plovdiv. This difference likely reflects local environmental conditions, including coastal influence and surface characteristics, which are known to enhance resuspension processes. Similar dust-related factors have been reported in coastal and Mediterranean regions, where both local resuspension and long-range transport contribute to particulate matter variability [30,31]. SOM clustering further supported this distinction by isolating patterns associated with particulate-dominated conditions in Varna.

The TSA results indicated that temporal behavior differed substantially among source types, even when their chemical signatures were similar. Traffic-related factors in both cities exhibited stronger seasonal components and more pronounced weekday–weekend differences, reflecting activity-driven variability, which is well documented in urban air quality studies [32]. In contrast, sulfur-rich combustion sources showed weaker weekly variability, suggesting more stable emission patterns. The crustal/resuspended dust factor in Varna was characterized by low seasonal strength but a significant weekday–weekend signal, indicating a distinct temporal signature compared to other sources.

Importantly, the observed trends in factor contributions should be interpreted with caution. Since PMF output represents relative contributions, the detected increases or decreases do not necessarily correspond to absolute changes in emissions. Instead, they reflect shifts in the relative importance of sources within the overall pollutant mixture. Similar limitations have been highlighted in recent methodological assessments, which emphasize that temporal variations in receptor model outputs may arise from changes in atmospheric conditions, source mixtures, or model structure rather than direct emission changes [33,34]. Therefore, the TSA results show how source contribution patterns have evolved, rather than providing definitive evidence of emission trends. It should also be noted that the observed temporal patterns may reflect not only changes in source contributions but also variations in meteorological conditions affecting pollutant dispersion.

Overall, the integration of SOM, PMF, and TSA demonstrates that, although the source types are broadly comparable between cities, their temporal signatures and relative contributions differ, emphasizing the importance of site-specific analysis. The added value of the proposed framework lies in its synergistic integration of pattern recognition, source apportionment, and temporal analysis, which enhances interpretability, reduces ambiguity in factor identification, and provides a more comprehensive basis for air quality assessment and management.

## 4. Materials and Methods

### 4.1. Sampling Site and Data Description

**Figure 8:**
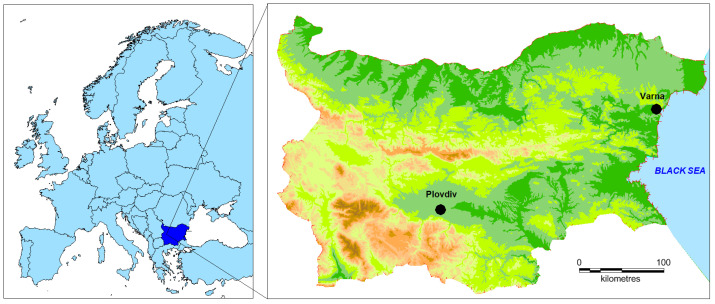
Geographical location of the urban background monitoring stations.

Plovdiv and Varna are the second- and third-most populated cities in Bulgaria. Plovdiv is located in southern Bulgaria in the Upper Thracian Plain, whereas Varna is situated in northeastern Bulgaria along the western Black Sea coast (Figure 8). The two cities represent distinct geographical settings, with Plovdiv characterized as an inland urban environment and Varna as a coastal urban environment.

According to official statistics from the National Statistical Institute of Bulgaria, the resident population within the administrative boundaries of Plovdiv ranged between approximately 338,000 and 347,000 inhabitants during 2009–2018, showing a slight overall increase over the studied period. Varna had a resident population between approximately 334,000 and 343,000 inhabitants during the same period, also exhibiting modest growth. Demographic conditions in both cities remained relatively stable throughout the investigated decade. During 2009–2018, both cities sustained established industrial activity. Plovdiv is a major inland industrial center with diversified manufacturing sectors, including machinery, electronics, metal processing, food production, and logistics, concentrated in large industrial zones such as the Trakia Economic Zone. Varna combines industrial production with maritime-related activities, including shipbuilding, port operations at the Port of Varna, and regional energy production facilities such as the Varna Thermal Power Plant.

Air quality data for the cities of Plovdiv and Varna were obtained from the National System for Environmental Monitoring operated by the Ministry of Environment and Water through the Executive Environment Agency of Bulgaria. Raw hourly measurements covering the period from 1 January 2009 to 31 December 2018 were collected for particulate matter with a diameter below 10 µm (PM_10_), nitrogen oxide (NO), nitrogen dioxide (NO_2_), sulfur dioxide (SO_2_), and carbon monoxide (CO). Data were retrieved from two automatic monitoring stations: Kamenitza in Plovdiv (24.765239° E, 42.142889° N), and Angel Kunchev in Varna (27.915733° E, 43.224389° N).

Both AMS sites operate as accredited automated background air quality monitoring facilities in accordance with the BDS EN ISO/IEC 17025 standard. This accreditation ensures strict quality assurance and quality control procedures, including routine three-month inspections, instrument maintenance, and calibration with certified standard gases. The raw hourly dataset underwent a structured cleaning procedure that included the removal of implausible measurements, instrument errors, and statistical outliers based on established validity criteria. The procedure includes the removal of negative values (excluding temperature) and the elimination of outliers using the three-sigma rule. Following quality control, daily mean concentrations were calculated and used for subsequent analyses. In addition to pollutant concentrations, corresponding daily records of ambient temperature, relative humidity, and wind speed were obtained for the same period and locations.

### 4.2. Data Elaboration

Basic statistical analysis was conducted using R (version 4.3.0) [35].

#### 4.2.1. Missing Data Imputation

The daily data were explored for missing values; the map of missing data was represented using the *vis_miss*() function in the visdat package (version 0.6.0) [36]. Missing values imputation was performed in the R software environment using the *na_seasplit*() function present in the imputeTS package (version 3.3) [37].

#### 4.2.2. SOM Analysis

SOM analysis and result representation were performed in the R software environment (version 4.0.5) [35] using the SOMEnv package (version 1.1.2) [38]. The SOM algorithm was initialized using the first two eigenvectors of the training dataset, and the number of neurons, map dimensions, and number of epochs were selected using heuristic rules related to the first two eigenvalues of the training dataset by Vesanto [25]. Vesanto suggested that the number of neurons to be used is five times the square root of the number of samples for a “regular” map. For training a more compressed map (“small”), the obtained number for a regular one has to be divided by four. For selecting the map dimensions, Vesanto suggested that their ratio has to be as close as possible to the square root of the ratio of the first two eigenvalues of the dataset. If the map is initialized using the first two eigenvectors of the dataset, the number of epochs can be calculated as fifty times the ratio between the number of neurons and the number of samples, while using at least two epochs. The map quality was assessed using quantization error (QE), topographic error (TE) and distribution match error (DME) [39].

#### 4.2.3. PMF Analysis

PMF analysis has been performed using EPA PMF software (version 5.0 https://www.epa.gov/air-research, accessed on 25 July 2025). Post-analytical processing, including the extraction of factor profiles and source contributions from PMF output files, was performed using the pmfr package (version0.3.003) [40] within the R statistical environment (version 4.3.0) [35]. PMF is a weighted least-squares approach based on a receptor model widely used in air pollution source apportionment. A complete overview of PMF is provided elsewhere [41,42]. PMF analysis was performed using daily average concentrations of the air pollutants as inputs. The uncertainties of the measurements were not explicitly investigated by the authors, so the uncertainty values for each measurement were calculated in accordance with the recommended procedure to encompass both analytical and sampling error [4]. Briefly, we reconstructed the uncertainty matrix using the fifth percentile of the concentration for each species as a proxy for the detection limit (*DL*) in the formula:

$$s_{ij}=\sqrt{{\left(error\;fraction\times C_{ij}\right)}^{2}+{\left(0.5\times DL\right)}^{2}}$$

Following this approach, when $C_{ij}>DL$, the uncertainty is dominated by the selected percentage of the concentration (in this case 20%); when $C_{ij}<DL$, the uncertainty is set to a fixed high value (four times *DL*) to reduce the point’s influence [43].

The two input matrices consisted of 3692 time units for 5 species. To converge on the global minimum of the objective function Q, 50 independent model runs were executed for each factor solution (ranging from 2 to 4 factors) using random starting seeds. The robustness of the final solution was evaluated using the Q_true_/Q_exp_ ratio and the distribution of scaled residuals, ensuring they fell predominantly within the ±3 range. The optimal number of factors was determined through a rigorous multi-criteria approach. This involved monitoring the change in Q_true_/Q_exp_ (dQ) as the number of factors increased, alongside the physical interpretability of the resulting source profiles. To quantify the statistical and rotational uncertainty of the chosen solution, bootstrap (BS) and displacement (DISP) were employed [43]. A total of 100 BS runs were performed to assess the stability of the factor profiles. A mapping threshold of >80% was required for a solution to be considered stable. DISP was used to explore rotational ambiguity by determining if the Q value changed significantly when factor profile elements were shifted. The solution was deemed robust if no factor swaps occurred and the dQ remained within the acceptable limits defined by the EPA PMF 5.0 guide [42].

#### 4.2.4. TSA

Time-series analysis was applied to the daily Positive Matrix Factorization (PMF) source contribution estimates for each identified factor at monitoring sites in Plovdiv and Varna. All analyses were conducted using the R statistical environment [35] and were performed separately for each PMF factor and city. Daily PMF contribution time series were constructed for the period 2009–2018. Seasonal–trend decomposition using Loess (STL) was applied to each daily PMF contribution time series to separate long-term trend, seasonal, and remainder components. STL decomposition was performed using the *stl*() function from the base stats package, with a periodic seasonal window and robust fitting to reduce sensitivity to extreme values. The resulting trend and seasonal components were used for subsequent statistical analyses and interpretation. The presence of monotonic long-term trends in PMF source contributions was assessed using the non-parametric Mann–Kendall test, implemented via the *mk.test*() function from the trend package (version 1.1.6) [44]. Trend magnitude was quantified using Sen’s slope estimator (*sens.slope*()), providing a robust estimate of the annual rate of change. Sen’s slope estimates were expressed as percentage change per year relative to the mean contribution level to facilitate comparison between sources and sites. Seasonality in PMF source contributions was evaluated using complementary statistical and descriptive approaches. Stable seasonality was tested using the QS test (*qs*() function) from the seastests package (version 0.15.4) [45]. In parallel, intra-annual variability was assessed using the Kruskal–Wallis test to examine differences in PMF contributions among months, treating month as a categorical factor. Seasonal strength was quantified as the proportion of variance attributable to the seasonal component relative to the combined seasonal and remainder variance, following established STL-based metrics. This measure provides a descriptive estimate of the relative importance of seasonality in each PMF source contribution time series. To investigate potential differences between anthropogenic activity patterns, weekday–weekend contrasts in PMF source contributions were examined. Days were classified as weekdays (Monday–Friday) or weekends (Saturday–Sunday). Differences between the two groups were tested using the non-parametric Wilcoxon rank-sum test (Mann–Whitney U test).

## 5. Conclusions

The added value of the proposed framework derives from the synergistic integration of SOM, PMF, and TSA, which collectively enhance source validation, interpretability, and robustness of the apportionment results.

The integrated approach substantially enhances policy relevance by coupling source attribution with temporal behavior and pattern classification. The framework allows policymakers to identify not only dominant pollution patterns but also the conditions and periods under which they exert the strongest influence. Such evidence directly supports targeted interventions, including seasonal emission control strategies, traffic management measures during stagnation episodes, and source-specific mitigation programs aligned with local pollution dynamics.

The applicability of the proposed methodological framework was demonstrated through its implementation on air quality datasets from two of the largest Bulgarian cities, highlighting its potential as a transferable analytical approach for urban environments characterized by complex emission mixtures and evolving anthropogenic pressures.

## Figures and Tables

**Table 1:** Summary of air pollutant concentrations (µg/m^3^) and meteorological variables, including Wilcoxon rank-sum test results.

Variable	Site	Missing Data N (%)	25th Percentile	Median	Mean	75th Percentile	Wilcoxon Test
CO	Plovdiv	9 (0.25)	0.29	0.43	0.57	0.69	*p* < 0.001
	Varna	16 (0.44)	0.24	0.36	0.46	0.62	
NO	Plovdiv	20 (0.55)	2.29	4.10	9.66	9.97	*p* < 0.05
	Varna	15 (0.41)	2.03	4.01	7.94	9.52	
NO_2_	Plovdiv	20 (0.55)	15.85	21.24	24.69	30.55	*p* < 0.001
	Varna	16 (0.44)	7.74	13.09	17.99	25.21	
PM_10_	Plovdiv	105 (2.88)	26.29	37.15	47.83	54.96	*p* < 0.001
	Varna	39 (1.07)	16.07	25.26	29.76	37.87	
SO_2_	Plovdiv	30 (0.82)	8.57	12.89	15.22	18.03	*p* < 0.001
	Varna	19 (0.52)	3.48	5.82	7.41	9.52	
Temperature (°C)	Plovdiv	2 (0.05)	6.93	14.43	14.09	21.95	ns
	Varna	13 (0.36)	7.15	13.84	13.81	21.39	
Relative humidity (%)	Plovdiv	2 (0.05)	45.66	60.48	61.88	76.87	*p* < 0.001
	Varna	14 (0.38)	65.86	73.09	73.22	80.97	
Wind speed (m/s)	Plovdiv	2 (0.05)	0.30	0.44	0.57	0.73	*p* < 0.001
	Varna	13 (0.36)	1.37	1.87	1.92	2.40	

ns = not significant.

**Table 2:** Summary of time-series characteristics of PMF factor contributions in Plovdiv and Varna.

City	Factor	Related Sources	Trend (% yr^−1^)	Trend Direction	Seasonal Strength (%)	Weekday–Weekend Signal
Plovdiv	F1	traffic (fresh exhaust)	+2.43	Increasing	33.77	Significant
	F2	sulfur-rich combustion	+4.64	Increasing	18.58	Not significant
	F3	traffic-related aerosol	−5.14	Decreasing	24.35	Significant
Varna	F1	sulfur-rich combustion	+2.97	Increasing	8.19	Not significant
	F2	crustal/resuspend-ed dust	−1.68	Decreasing	6.46	Significant
	F3	traffic-related aerosol	−9.30	Decreasing	25.20	Significant
	F4	traffic (fresh exhaust)	1.03	Increasing	27.21	Significant

## Data Availability

Data are available upon request from the Bulgarian Ministry of Environment and Water. The datasets used in this study were obtained by the research team from the Ministry under official request procedures.

## Supplementary Materials

The following supporting information can be downloaded at: https://www.mdpi.com/article/10.3390/molecules31101725/s1, Table S1: Summary of annual mean PM_10_ exceedances of the EU limit (40 µg/m^3^), with non-compliant years shown in bold, Table S2: Summary of daily mean PM_10_ exceedances of the EU limit (50 µg/m^3^), with non-compliant years (>35 per year) shown in bold, Table S3: Summary of annual mean NO_2_ exceedances of the EU limit (40 µg/m^3^), with non-compliant years shown in bold, Table S4. Summary of daily mean NO_2_ exceedances of the WHO limit (25 µg/m^3^), Table S5: Summary of daily mean SO_2_ exceedances of the WHO limit (40 µg/m^3^), Figure S1: Missing data distribution of the whole dataset, Figure S2: Neuron number assignment, Table S6: Basic statistics of modeled values for pollutants, Table S7: Percentage of samples for each site represented by each neuron, Table S8: Statistical evaluation of PMF model source apportionment factors for the two datasets, Figure S3: Profiles of the three PMF factors for the Plovdiv dataset, Figure S4: Profiles of the four PMF factors for the Varna dataset, Figure S5: Time-series decomposition of PMF Factor 1 contributions in Varna, including long-term trend, seasonal component, and residuals, Figure S6: Time-series decomposition of PMF Factor 2 contributions in Varna, including long-term trend, seasonal component, and residuals, Figure S7: Time-series decomposition of PMF Factor 3 contributions in Varna, including long-term trend, seasonal component, and residuals, Figure S8: Time-series decomposition of PMF Factor 4 contributions in Varna, including long-term trend, seasonal component, and residuals.

## Abbreviations

The following abbreviations are used in this manuscript:

SOM	Self-Organizing Map
PMF	Positive Matrix Factorization
TSA	Time-Series Analysis
AMS	Automatic Monitoring Station
