# Supplementary material for: Integrating Self-Organizing Maps, Positive Matrix Factorization and Time-Series Decomposition for Urban Air Pollution Source Apportionment: A Comparative Study of Bulgarian Cities
Source: Molecules. 2026 May 19;31(10):1725. doi: 10.3390/molecules31101725 (PMC13209328; doi:10.3390/molecules31101725)
Supplement: Supplementary file 1 [file molecules-31-01725-s001.zip › molecules-4230633-supplementary.pdf]

SUPPLEMENTARY MATERIAL

Integrating Self-Organizing Maps, Positive Matrix Factorization and Time-Series Decomposition for Urban Air Pollution Source Apportionment: A Comparative Study of Bulgarian Cities

- Table S1. Summary of annual mean PM10 exceedances of the EU limit (40 μg/m^3), with non-compliant years shown in bold
- Table S2 Summary of daily mean PM10 exceedances of the EU limit (50 μg/m^3), with non-compliant years (>35 per year) shown in bold
- Table S3. Summary of annual mean NO_2 exceedances of the EU limit (40 μg/m^3), with non-compliant years shown in bold
- Table S4. Summary of daily mean NO_2 exceedances of the WHO limit (25 μg/m^3)
- Table S5. Summary of daily mean SO_2 exceedances of the WHO limit (40 μg/m^3)
- Figure S1: Missing data distribution of the whole dataset
- Figure S2: Neuron number assignment
- Table S6: Basic statistics of modelled values for pollutants
- Table S7: Percentage of samples for each site represented by each neuron
- Table S8: Statistical evaluation of PMF model source apportionment factors for the two datasets.
- Figure S3. Profiles of the three PMF factors for the Plovdiv dataset.
- Figure S4. Profiles of the three PMF factors for the Varna dataset.
- Figure S5. Time-series decomposition of PMF Factor 1 contributions in Varna, including long-term trend, seasonal component, and residuals.
- Figure S6. Time-series decomposition of PMF Factor 2 contributions in Varna, including long-term trend, seasonal component, and residuals.
- Figure S7. Time-series decomposition of PMF Factor 3 contributions in Varna, including long-term trend, seasonal component, and residuals.
- Figure S8. Time-series decomposition of PMF Factor 4 contributions in Varna, including long-term trend, seasonal component, and residuals.

Table S1. Summary of annual mean PM10 exceedances of the EU limit (40 μg/m^3), with non-compliant years shown in bold

Site	Year	mean
Plovdiv	**2009**	57.79
	**2010**	54.40
	**2011**	60.54
	**2012**	55.13
	**2013**	54.88
	2014	34.90
	2015	37.93
	**2016**	40.93
	**2017**	43.29
	2018	37.45
Varna	2009	34.12
	2010	37.79
	2011	33.01
	2012	23.60
	2013	24.31
	2014	35.71
	2015	27.70
	2016	29.76
	2017	25.39
	2018	26.34

Table S2 Summary of daily mean PM10 exceedances of the EU limit (50 μg/m³), with non-compliant years (>35 per year) shown in bold

**Site**	**Year**	**days exceed**
Plovdiv	**2009**	162
	**2010**	134
	**2011**	152
	**2012**	129
	**2013**	132
	**2014**	44
	**2015**	65
	**2016**	81
	**2017**	82
	**2018**	72
Varna	**2009**	58
	**2010**	87
	**2011**	70
	2012	30
	2013	29
	**2014**	76
	2015	33
	2016	35
	2017	25
	2018	28

Table S3. Summary of annual mean NO_2 exceedances of the EU limit (40 μg/m^3), with non-compliant years shown in bold

**Site**	**Year**	**mean**
Plovdiv	2009	27.15
	2010	26.60
	2011	24.41
	2012	26.53
	2013	24.14
	2014	22.12
	2015	24.23
	2016	25.52
	2017	25.75
	2018	20.23
Varna	2009	29.68
	2010	28.77
	2011	25.85
	2012	10.98
	2013	4.36
	2014	9.69
	2015	13.87
	2016	8.59
	2017	15.22
	2018	32.98

Table S4. Summary of daily mean NO_2 exceedances of the WHO limit (25 µg/m^3)

Site	Year	days exceed
Plovdiv	2009	168
	2010	154
	2011	130
	2012	152
	2013	134
	2014	115
	2015	128
	2016	151
	2017	132
	2018	76
Varna	2009	205
	2010	186
	2011	183
	2012	51
	2013	0
	2014	30
	2015	36
	2016	0
	2017	25
	2018	202

Table S5. Summary of daily mean SO_2 exceedances of the WHO limit (40 μg/m^3)

Site	Year	days exceed
Plovdiv	2009	25
	2010	22
	2011	56
	2012	26
	2013	7
	2014	14
	2015	0
	2016	0
	2017	0
	2018	0
Varna	2009	7
	2010	1
	2011	6
	2012	0
	2013	1
	2014	0
	2015	0
	2016	0
	2017	0
	2018	0

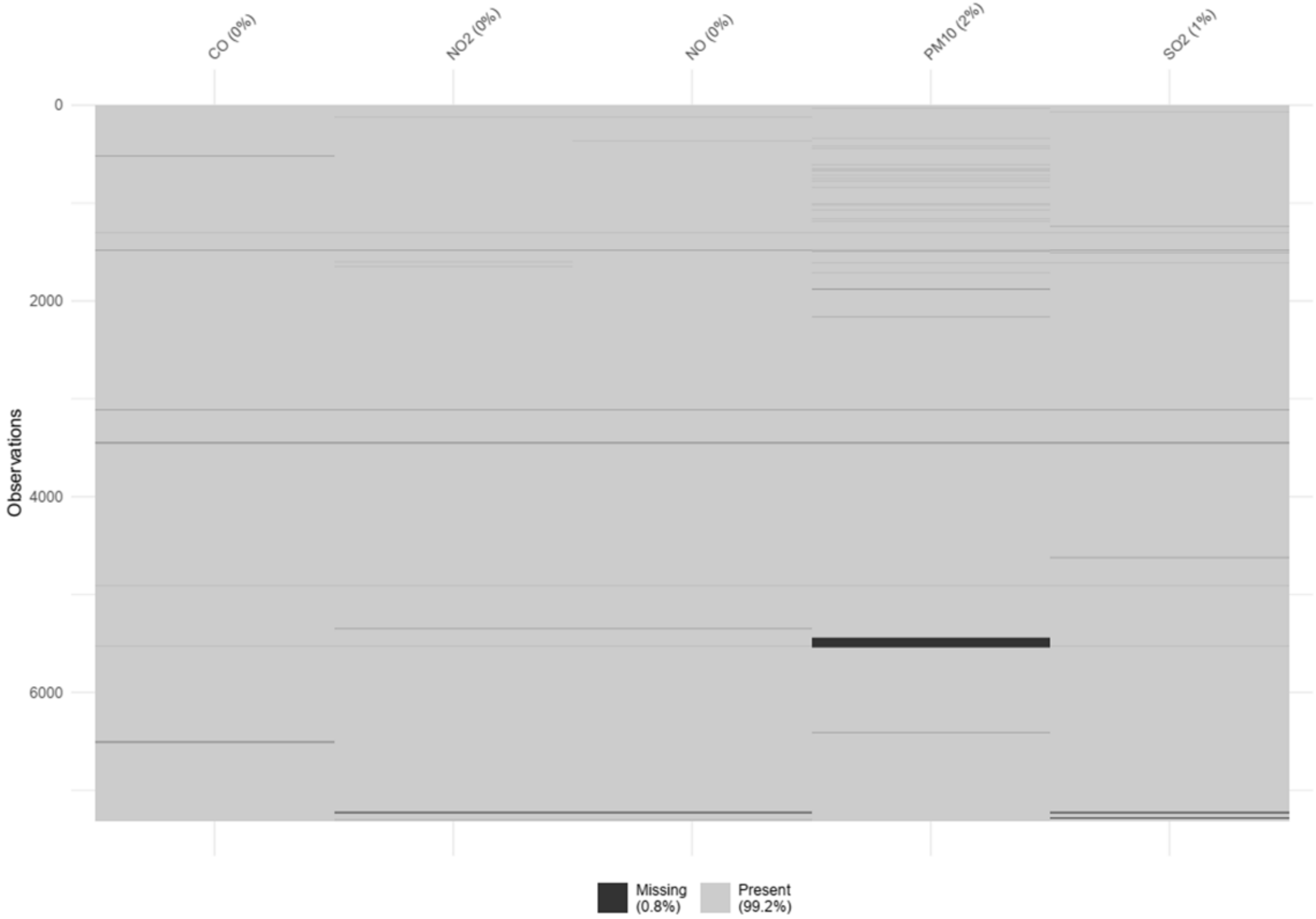

Figure S1. Missing data distribution of the whole dataset.

Table S6 Basic statistics of modelled values for pollutants (values expressed in μg/m³) and meteorological variables (values expressed in °C, % and m/s respectively). §Value that is (1.5*Interquartile Range) far from 1stQuartile; #Value that is (1.5*Interquartile Range) far from 3rdQuartile

Statistic	CO	NO2	NO	PM10	SO2	Temp	UMR	Wind
Min	0.2	15.4	2.3	21.6	4	1	35	0.4
LowerOut	0.2	15.4	2.3	21.6	4	1	45	0.4
1stQ	0.3	18.6	5.0	26.1	8	7	65	0.6
Median	0.4	21.8	6.6	33.4	10	10	75	0.9
3rdQ	0.7	30.9	12.9	45.3	15	20	79	1.6
UpperOut	1.2	48.8	24.4	73.5	24	25	89	2.7
Max	1.8	55.2	50.0	159.0	43	25	89	2.7

Figure S2. Neuron number assignment

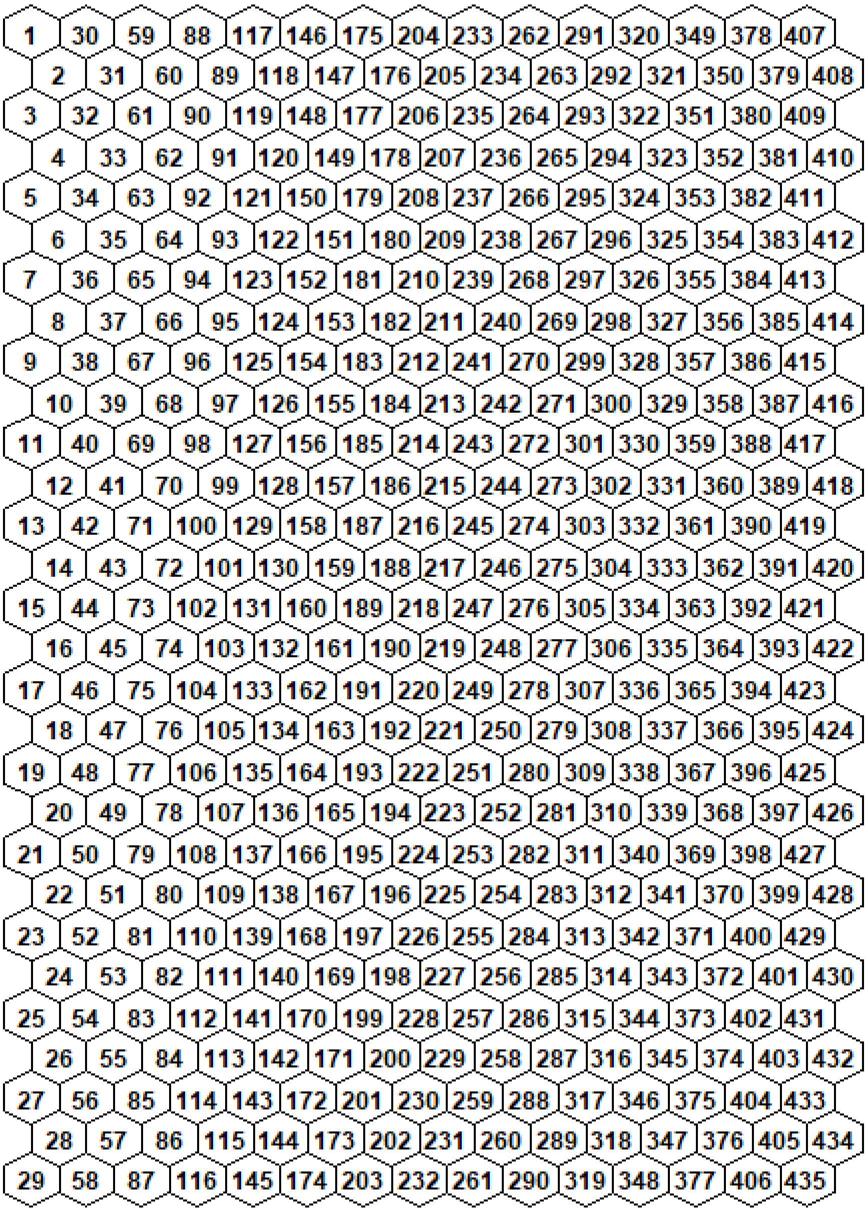

Table S7 Percentage of samples for each site represented by each neuron (refer to Figure S2 for neuron number assignment)

Neuron	PL (%)	VA (%)	Neuron	PL (%)	VA (%)	Neuron	PL (%)	VA (%)	Neuron	PL (%)	VA (%)	Neuron	PL (%)	VA (%)	Neuron	PL (%)	VA (%)	Neuron	PL (%)	VA (%)
**1**	100	0	**30**	100	0	**59**	100	0	**88**	100	0	**117**	92	8	**146**	59	41	**175**	6	94
**2**	100	0	**31**	100	0	**60**	100	0	**89**	100	0	**118**	63	38	**147**	30	70	**176**	0	100
**3**	100	0	**32**	100	0	**61**	100	0	**90**	100	0	**119**	75	25	**148**	20	80	**177**	0	100
**4**	100	0	**33**	100	0	**62**	100	0	**91**	100	0	**120**	17	83	**149**	0	100	**178**	0	100
**5**	100	0	**34**	100	0	**63**	100	0	**92**	100	0	**121**	20	80	**150**	0	100	**179**	0	100
**6**	100	0	**35**	100	0	**64**	100	0	**93**	94	6	**122**	50	50	**151**	0	100	**180**	0	100
**7**	100	0	**36**	100	0	**65**	100	0	**94**	96	4	**123**	100	0	**152**	0	100	**181**	0	100
**8**	100	0	**37**	100	0	**66**	100	0	**95**	100	0	**124**	94	6	**153**	17	83	**182**	0	100
**9**	100	0	**38**	100	0	**67**	100	0	**96**	100	0	**125**	93	7	**154**	78	22	**183**	9	91
**10**	100	0	**39**	100	0	**68**	100	0	**97**	100	0	**126**	100	0	**155**	100	0	**184**	80	20
**11**	100	0	**40**	100	0	**69**	100	0	**98**	100	0	**127**	100	0	**156**	100	0	**185**	82	18
**12**	100	0	**41**	100	0	**70**	100	0	**99**	100	0	**128**	100	0	**157**	100	0	**186**	100	0
**13**	100	0	**42**	80	20	**71**	100	0	**100**	100	0	**129**	100	0	**158**	100	0	**187**	100	0
**14**	100	0	**43**	100	0	**72**	100	0	**101**	100	0	**130**	88	13	**159**	100	0	**188**	88	13
**15**	100	0	**44**	100	0	**73**	71	29	**102**	90	10	**131**	100	0	**160**	89	11	**189**	100	0
**16**	94	6	**45**	88	13	**74**	60	40	**103**	75	25	**132**	46	54	**161**	67	33	**190**	89	11
**17**	96	4	**46**	100	0	**75**	50	50	**104**	69	31	**133**	60	40	**162**	86	14	**191**	78	22
**18**	89	11	**47**	78	22	**76**	89	11	**105**	55	45	**134**	40	60	**163**	92	8	**192**	100	0
**19**	93	7	**48**	100	0	**77**	88	13	**106**	84	16	**135**	92	8	**164**	100	0	**193**	100	0
**20**	97	3	**49**	80	20	**78**	90	10	**107**	93	7	**136**	93	7	**165**	100	0	**194**	100	0
**21**	85	15	**50**	100	0	**79**	78	22	**108**	100	0	**137**	100	0	**166**	100	0	**195**	83	17
**22**	83	17	**51**	100	0	**80**	100	0	**109**	100	0	**138**	100	0	**167**	100	0	**196**	100	0
**23**	93	7	**52**	100	0	**81**	75	25	**110**	100	0	**139**	80	20	**168**	83	17	**197**	100	0
**24**	97	3	**53**	0	0	**82**	78	22	**111**	75	25	**140**	100	0	**169**	100	0	**198**	100	0
**25**	87	13	**54**	100	0	**83**	89	11	**112**	100	0	**141**	78	22	**170**	100	0	**199**	90	10

Neuron	PL (%)	VA (%)	Neuron	PL (%)	VA (%)	Neuron	PL (%)	VA (%)	Neuron	PL (%)	VA (%)	Neuron	PL (%)	VA (%)	Neuron	PL (%)	VA (%)	Neuron	PL (%)	VA (%)
**26**	89	11	**55**	83	17	**84**	83	17	**113**	89	11	**142**	89	11	**171**	100	0	**200**	85	15
**27**	93	7	**56**	88	13	**85**	78	22	**114**	71	29	**143**	94	6	**172**	81	19	**201**	88	13
**28**	100	0	**57**	100	0	**86**	100	0	**115**	88	13	**144**	100	0	**173**	100	0	**202**	100	0
**29**	100	0	**58**	100	0	**87**	95	5	**116**	70	30	**145**	84	16	**174**	100	0	**203**	70	30
**233**	0	100	**262**	0	100	**291**	0	100	**320**	0	100	**349**	0	100	**378**	0	100	**407**	0	100
**234**	0	100	**263**	0	100	**292**	0	100	**321**	0	100	**350**	0	100	**379**	0	100	**408**	0	100
**235**	0	100	**264**	0	100	**293**	0	100	**322**	0	100	**351**	0	100	**380**	0	100	**409**	0	100
**236**	0	100	**265**	0	100	**294**	0	100	**323**	0	100	**352**	0	100	**381**	0	100	**410**	4	96
**237**	0	100	**266**	0	100	**295**	0	100	**324**	0	100	**353**	0	100	**382**	0	100	**411**	0	100
**238**	0	100	**267**	0	100	**296**	0	100	**325**	0	100	**354**	0	100	**383**	0	100	**412**	0	100
**239**	0	100	**268**	0	100	**297**	0	100	**326**	0	100	**355**	0	100	**384**	0	100	**413**	0	100
**240**	0	100	**269**	0	100	**298**	0	100	**327**	0	100	**356**	0	100	**385**	0	100	**414**	3	97
**241**	0	100	**270**	0	100	**299**	0	100	**328**	0	100	**357**	0	100	**386**	0	100	**415**	2	98
**242**	13	87	**271**	0	100	**300**	18	82	**329**	8	92	**358**	6	94	**387**	0	100	**416**	0	100
**243**	11	89	**272**	20	80	**301**	11	89	**330**	0	100	**359**	0	100	**388**	10	90	**417**	3	97
**244**	9	91	**273**	18	82	**302**	8	92	**331**	0	100	**360**	14	86	**389**	0	100	**418**	2	98
**245**	14	86	**274**	5	95	**303**	0	100	**332**	7	93	**361**	20	80	**390**	60	40	**419**	6	94
**246**	38	63	**275**	9	91	**304**	12	88	**333**	6	94	**362**	0	100	**391**	29	71	**420**	23	77
**247**	60	40	**276**	58	42	**305**	10	90	**334**	0	100	**363**	33	67	**392**	0	100	**421**	11	89
**248**	100	0	**277**	63	38	**306**	59	41	**335**	8	92	**364**	13	88	**393**	0	100	**422**	0	100
**249**	100	0	**278**	77	23	**307**	64	36	**336**	25	75	**365**	14	86	**394**	50	50	**423**	0	100
**250**	100	0	**279**	100	0	**308**	100	0	**337**	40	60	**366**	20	80	**395**	14	86	**424**	0	100
**251**	100	0	**280**	100	0	**309**	67	33	**338**	14	86	**367**	22	78	**396**	0	100	**425**	7	93
**252**	100	0	**281**	67	33	**310**	40	60	**339**	20	80	**368**	6	94	**397**	0	100	**426**	0	100
**253**	100	0	**282**	100	0	**311**	20	80	**340**	6	94	**369**	0	100	**398**	0	100	**427**	0	100
**254**	100	0	**283**	100	0	**312**	30	70	**341**	6	94	**370**	0	100	**399**	0	100	**428**	0	100
**255**	92	8	**284**	100	0	**313**	56	44	**342**	14	86	**371**	0	100	**400**	0	100	**429**	0	100
**256**	100	0	**285**	79	21	**314**	70	30	**343**	0	100	**372**	0	100	**401**	0	100	**430**	0	100

Neuron	PL (%)	VA (%)	Neuron	PL (%)	VA (%)	Neuron	PL (%)	VA (%)	Neuron	PL (%)	VA (%)	Neuron	PL (%)	VA (%)	Neuron	PL (%)	VA (%)	Neuron	PL (%)	VA (%)
**257**	83	17	**286**	77	23	**315**	71	29	**344**	0	100	**373**	0	100	**402**	0	100	**431**	0	100
**258**	80	20	**287**	93	7	**316**	67	33	**345**	14	86	**374**	0	100	**403**	0	100	**432**	0	100
**259**	83	17	**288**	92	8	**317**	53	47	**346**	38	63	**375**	0	100	**404**	0	100	**433**	0	100
**260**	75	25	**289**	100	0	**318**	78	22	**347**	23	77	**376**	0	100	**405**	0	100	**434**	0	100
**261**	52	48	**290**	80	20	**319**	53	47	**348**	14	86	**377**	0	100	**406**	0	100	**435**	0	100

Note: each neuron equals to 100 %

Table S8. Statistical evaluation of PMF model source apportionment factors for the two datasets.

Plovdiv			
**Diagnostic Test**	**2 Factors**	**3 Factors**	**4 Factors**
**Q/Qexp**	1.55	0.94	0.88
**BS Mapping**	100% for all factors	100% for all factors	70% for one factor
**DISP Swaps**	None	None	None
**Residuals**	Random (-3.88 to 4.33)	Random (-2.87 to 3.79)	Random (-1.91 to 4.07)
Varna			
**Diagnostic Test**	**2 Factors**	**3 Factors**	**4 Factors**
**Q/Qexp**	3.49 (Too high)	2.60 (Good)	2.06 (Better)
**BS Mapping**	100% for all factors	95% for one factor	100% for all factors
**DISP Swaps**	None	None	None
**Residuals**	Random (-3.79 to 4.85)	Random (-3.63 to 4.28)	Random (-3.25 to 3.51)

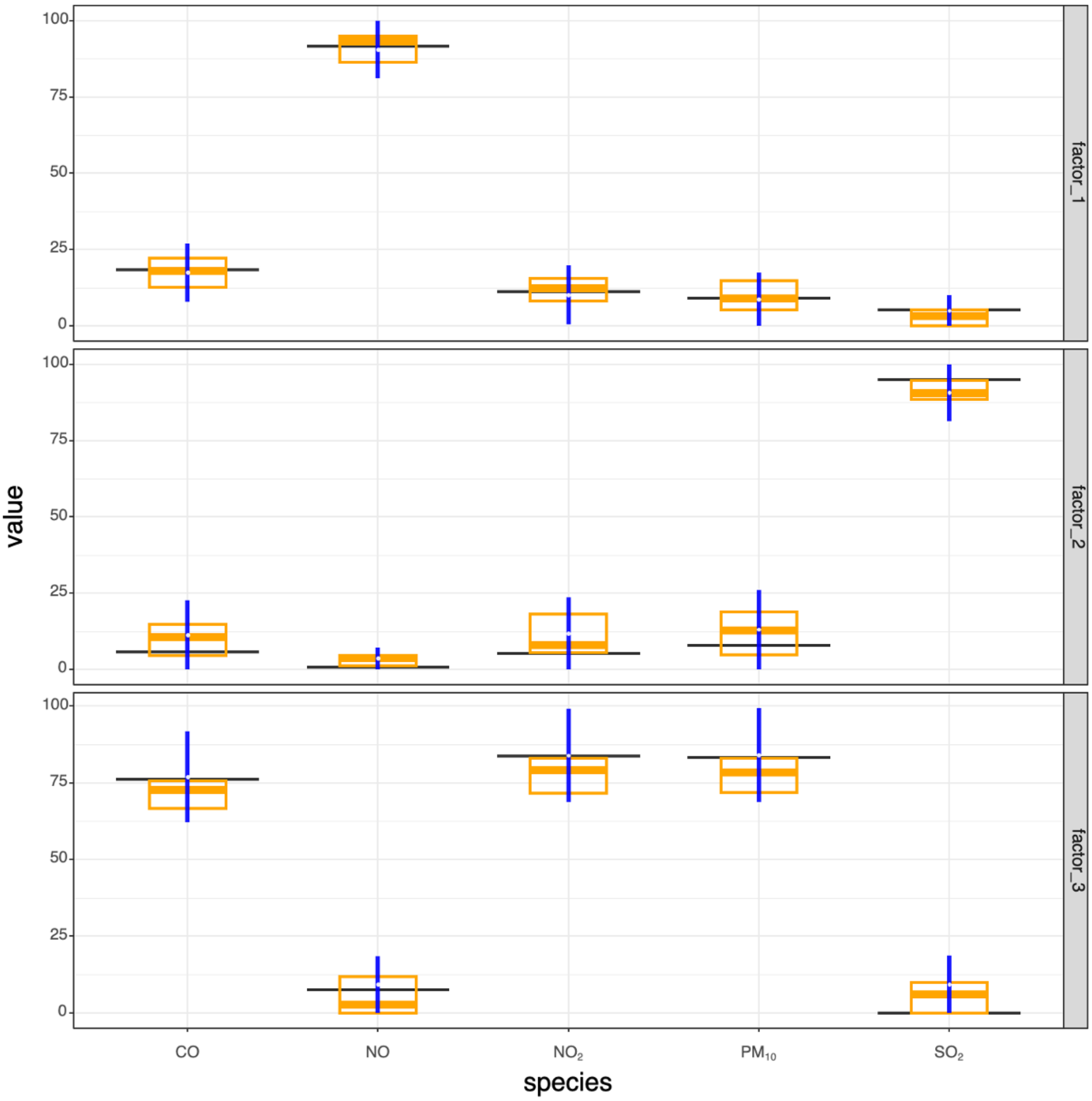

Figure S3. Profiles of the three PMF factors for the Plovdiv dataset. The intervals represent the DISP intervals. Black bars are the base values. The white circles are the mean fractional displacement (DISP) values, the asymmetric blue error bars represent the maximum and minimum DISP values. The orange boxes represent the uncertainty estimation based on the bootstrapping (BS) results (central line = median; box = interquartile range; n =100).

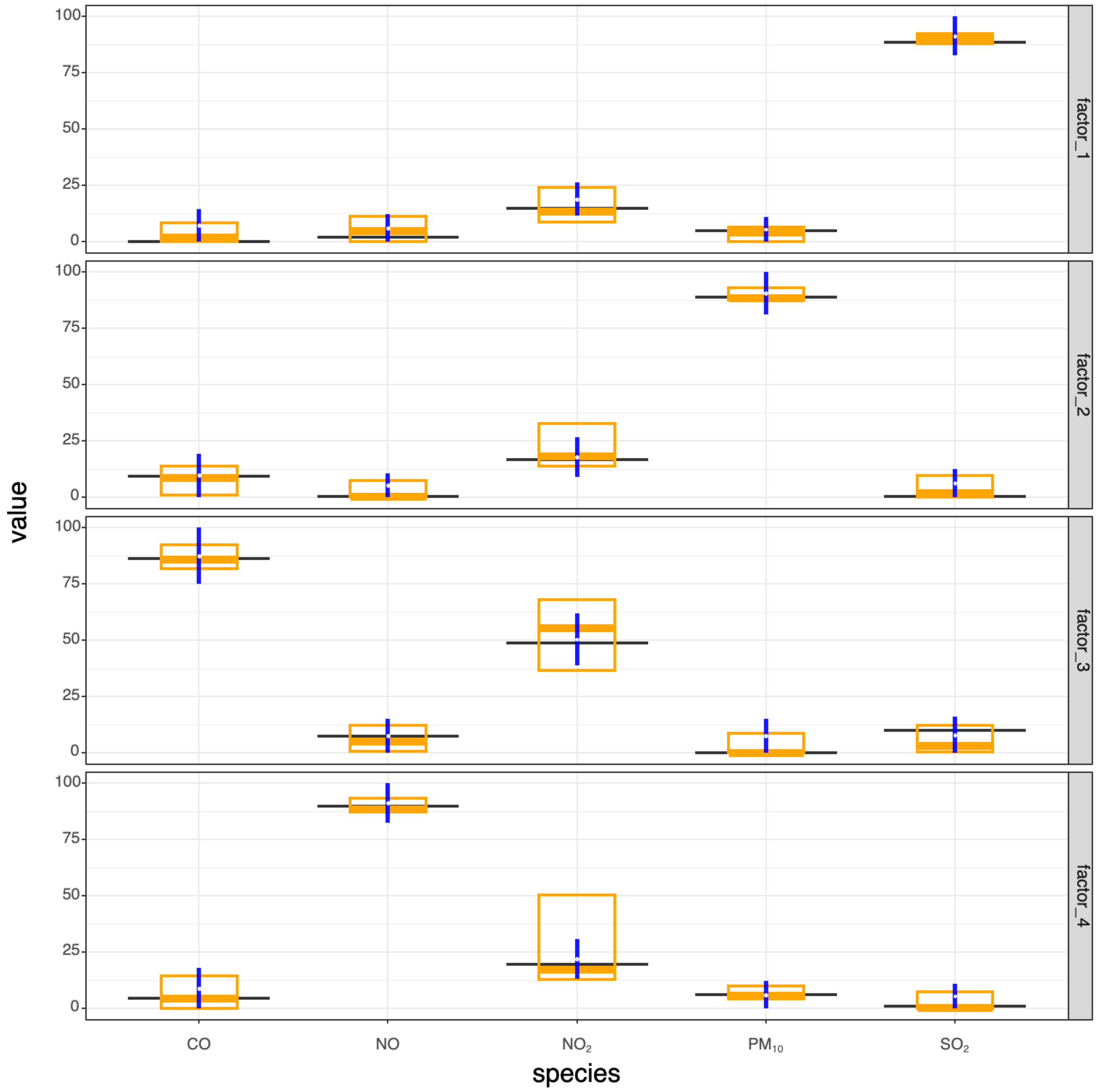

Figure S4. Profiles of the four PMF factors for the Varna dataset. The intervals represent the DISP intervals. Black bars are the base values. The white circles are the mean fractional displacement (DISP) values, the asymmetric blue error bars represent the maximum and minimum DISP values. The orange boxes represent the uncertainty estimation based on the bootstrapping (BS) results (central line = median; box = interquartile range; n =100).

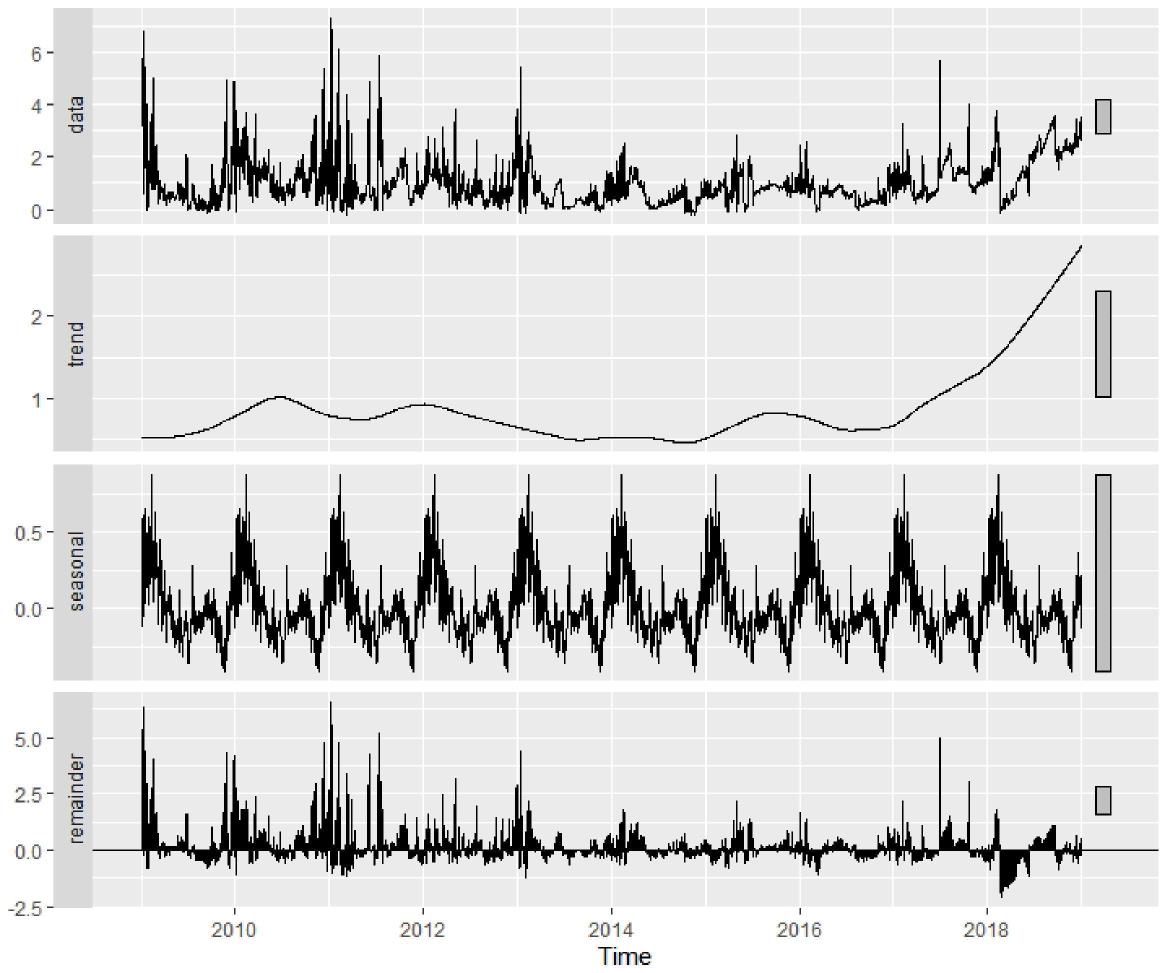

Figure S5 Time-series decomposition of PMF Factor 1 contributions in Varna, including long-term trend, seasonal component, and residuals.

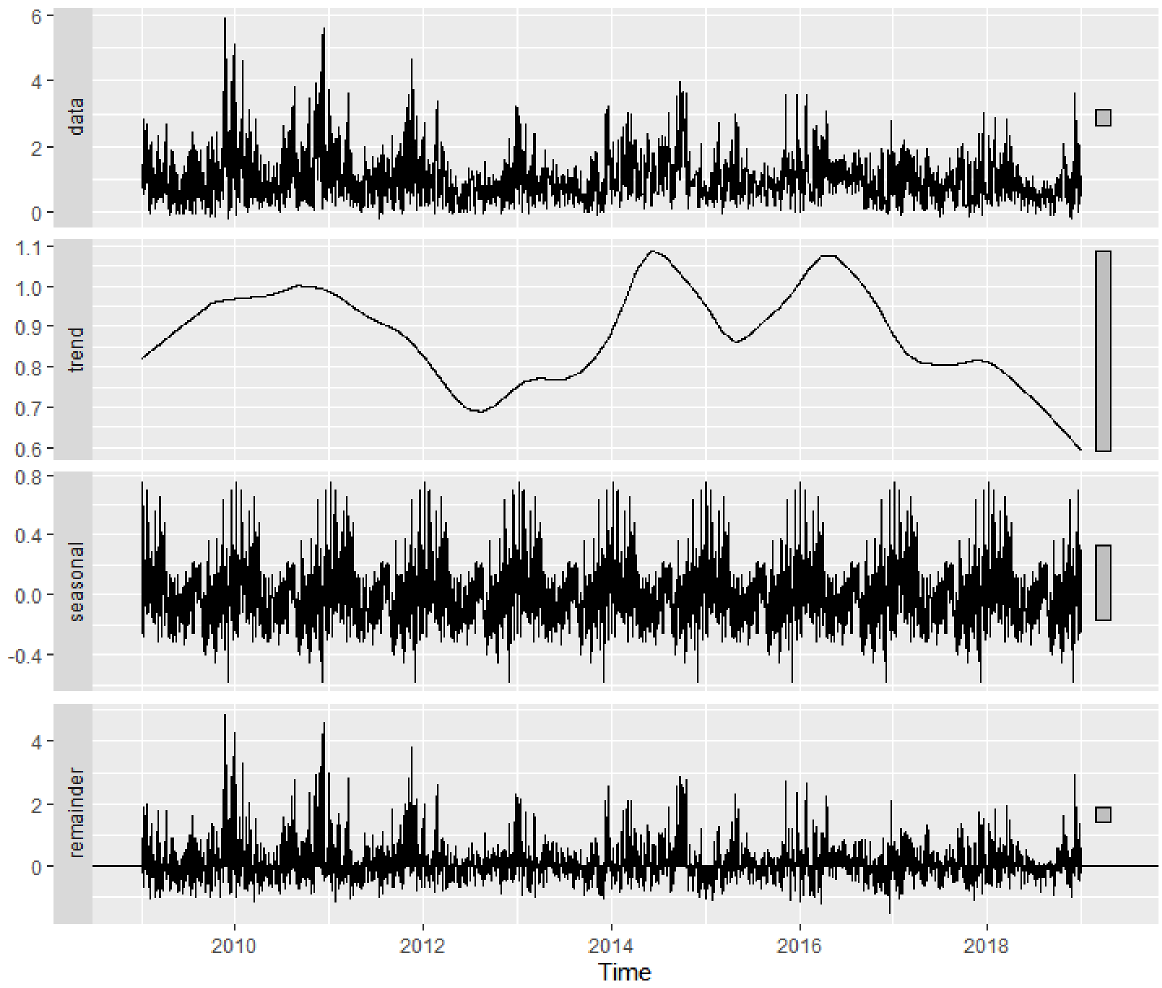

Figure S6 Time-series decomposition of PMF Factor 2 contributions in Varna, including long-term trend, seasonal component, and residuals.

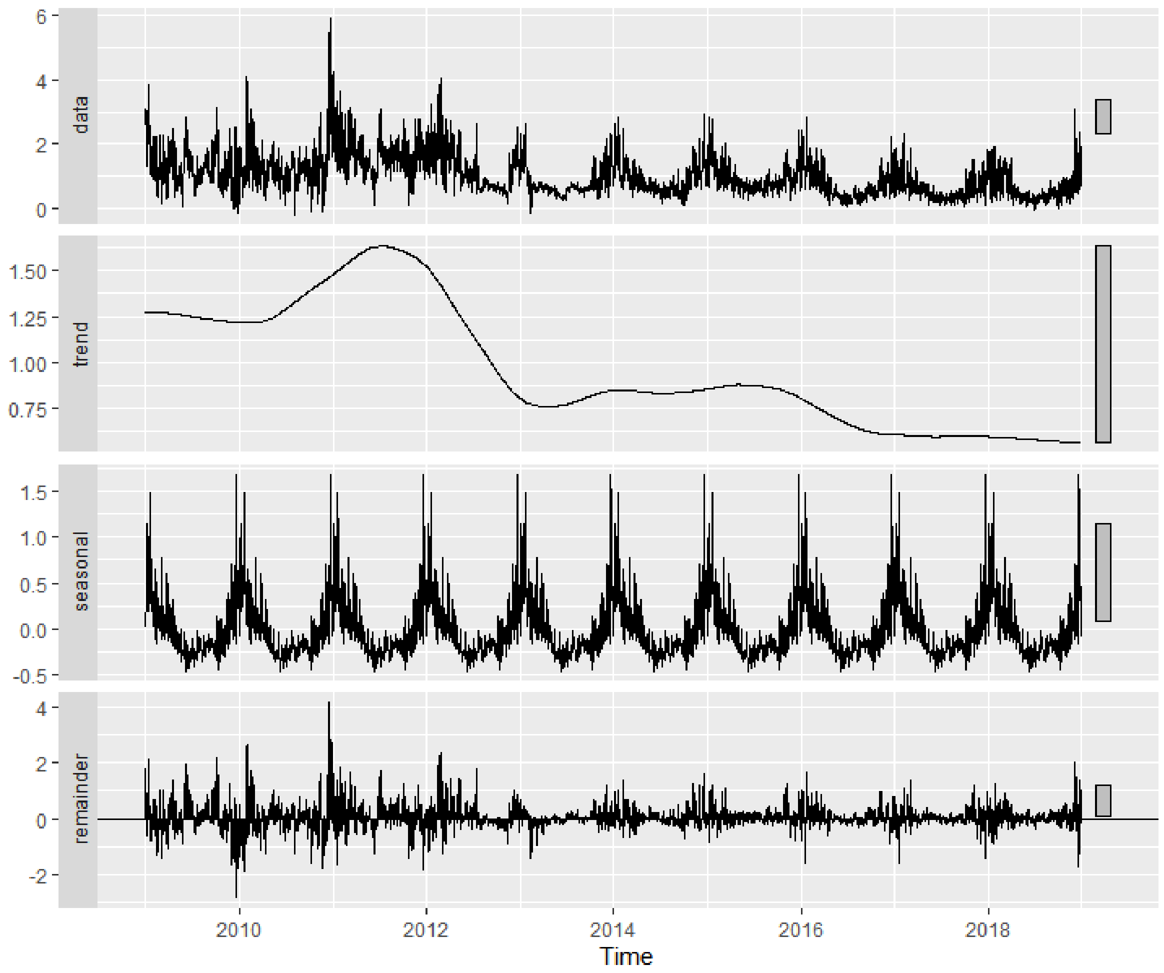

Figure S7 Time-series decomposition of PMF Factor 3 contributions in Varna, including long-term trend, seasonal component, and residuals.

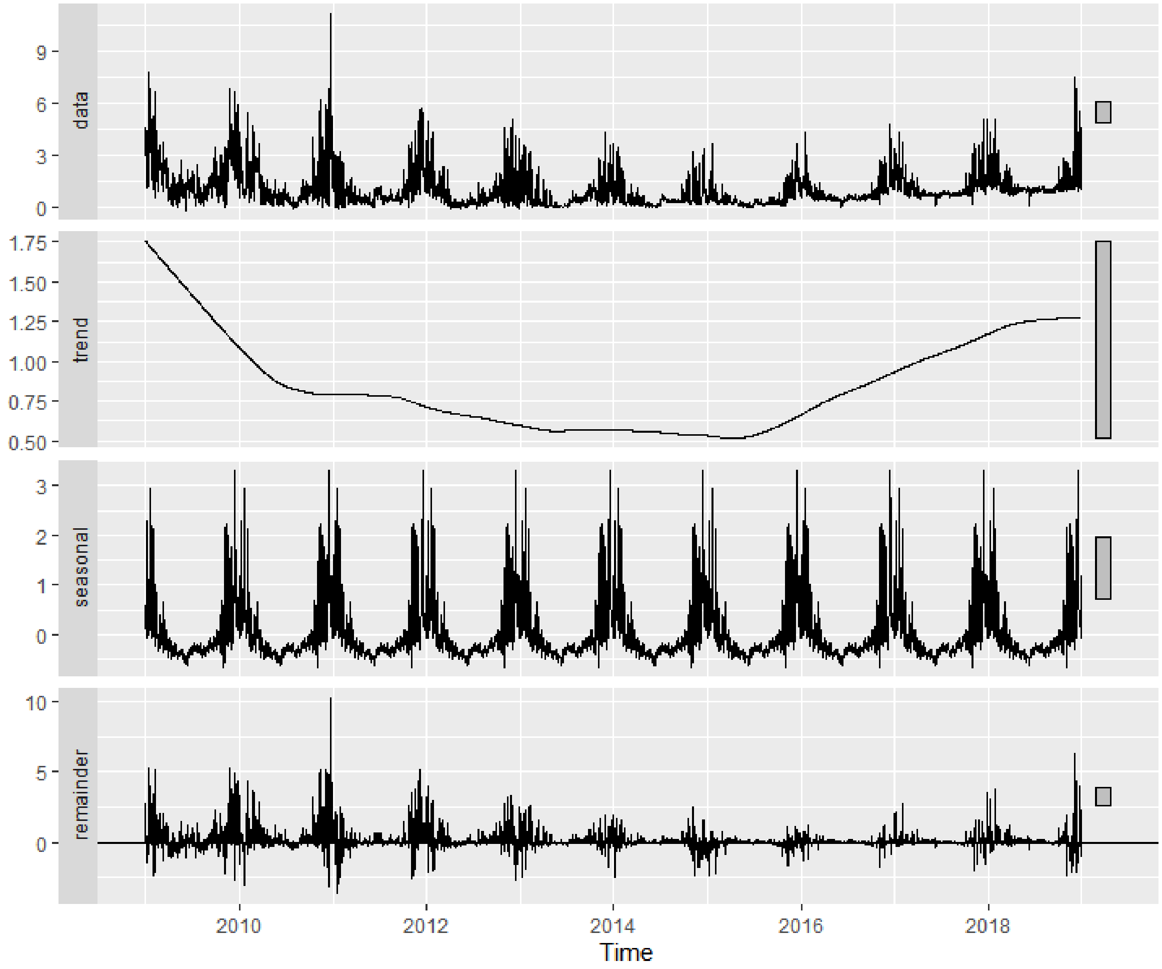

Figure S8 Time-series decomposition of PMF Factor 4 contributions in Varna, including long-term trend, seasonal component, and residuals.
